# 2770. Investigating the Impact of Non-CTX-M Producing ESBL-E Bloodstream Infections on Patient Outcomes

**DOI:** 10.1093/ofid/ofad500.2381

**Published:** 2023-11-27

**Authors:** Dariusz A Hareza, Patricia J Simner, Suiyini Fiawoo, Jae Hyoung Lee, Yehudit Bergman, Emily B Jacobs, Sara E Cosgrove, Pranita Tamma

**Affiliations:** Johns Hopkins University, Baltimore, Maryland; Johns Hopkins School of Medicine, Baltimore, MD; Johns Hopkins School of Medicine, Baltimore, MD; Johns Hopkins, Baltimore, Maryland; Johns Hopkins, Baltimore, Maryland; Johns Hopkins School of Medicine, Baltimore, MD; Johns Hopkins School of Medicine, Baltimore, MD; Johns Hopkins School of Medicine, Baltimore, MD

## Abstract

**Background:**

Studies investigating optimal treatment and patient outcomes for extended-spectrum β-lactamase-producing Enterobacterales (ESBL-E) generally do not distinguish between ESBL genes in bloodstream infections (BSI). To address a key knowledge gap, we compare the clinical outcomes of patients with non-CTX-M and CTX-M-producing ESBL-E BSIs.

**Methods:**

We collected 500 ceftriaxone-resistant Enterobacterales bloodstream isolates from patients in the Johns Hopkins Health System between 2018-2021. Broth microdilution confirmed antimicrobial susceptibilities, and whole genome sequencing identified ESBL genes. We used 1:3 propensity score matching (PSM) to compare the odds of 30-day mortality between patients infected with non-CTX-M and CTX-M ESBL-Es. Propensity scores were derived using seven variables: age, sex, severe immune compromise, Pitt bacteremia score ≥4, Charlson comorbidity index ≥5, adequate source control, and carbapenem use.

**Results:**

ESBL genes were confirmed in 396 isolates, including 370 (93%) *bla*_CTX-M_ genes and 26 (7%) non-*bla*_CTX-M_ ESBL genes: *bla*_SHV_ (13), *bla*_OXY_ (8), *bla*_VEB_ (5). Common species harboring *bla*_CTX-M_ were: *Escherichia coli* (265), *Klebsiella pneumoniae* (88), *K. oxytoca* (5), and *Enterobacter cloacae* (5); and non-*bla*_CTX-M_ were: *K. pneumoniae* (8), *K. oxytoca* (8), and *Proteus mirabilis* (5). After PSM, baseline patient characteristics were similar, and the aOR of 30-day mortality was 0.94 (95% CI 0.89-0.99) comparing patients with non-*bla*_CTX-M_ and *bla*_CTX-M_ isolates. In an exploratory analysis limited to patients with non-*bla*_CTX-M_ isolates: 2 of 15 patients (13%) receiving meropenem and 6 of 11 (55%) not receiving meropenem died within 30 days (aOR=0.61; 95% CI 0.42-0.90).

**Conclusion:**

Mortality was slightly lower in patients with BSIs due to non-CTX-M-producing ESBL-Es compared to CTX-M-producing ESBL-Es. Similar to what has been observed with CTX-M-producing ESBL-Es, meropenem is protective against mortality in patients infected with non-CTX-M producing ESBL-Es, underscoring the importance of diagnostics to detect a comprehensive array of ESBL genes among diverse Enterobacterales.

**Disclosures:**

**Patricia J. Simner, PhD**, Affinity Biosensors: Grant/Research Support|BD Diagnostics: Advisor/Consultant|BD Diagnostics: Grant/Research Support|Entasis: Advisor/Consultant|GeneCapture: Stocks/Bonds|Merck: Advisor/Consultant|OpGen Inc: Board Member|OpGen Inc: Grant/Research Support|OpGen Inc: Honoraria|Qiagen Sciences Inc: Advisor/Consultant|Qiagen Sciences Inc: Grant/Research Support|Shionogi Inc: Advisor/Consultant|T2 Biosystems: Grant/Research Support **Sara E. Cosgrove, MD, MS**, Debiopharm: Advisor/Consultant|Duke Clinical Research Institute: Advisor/Consultant

